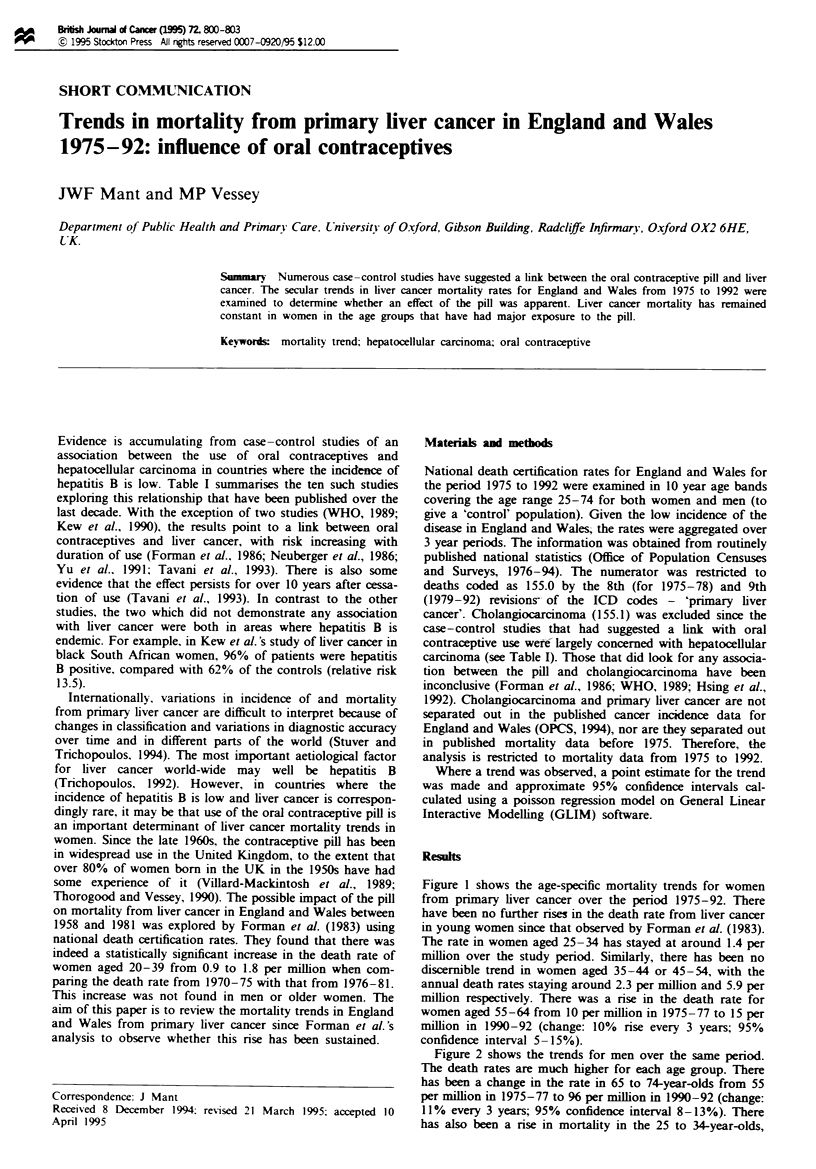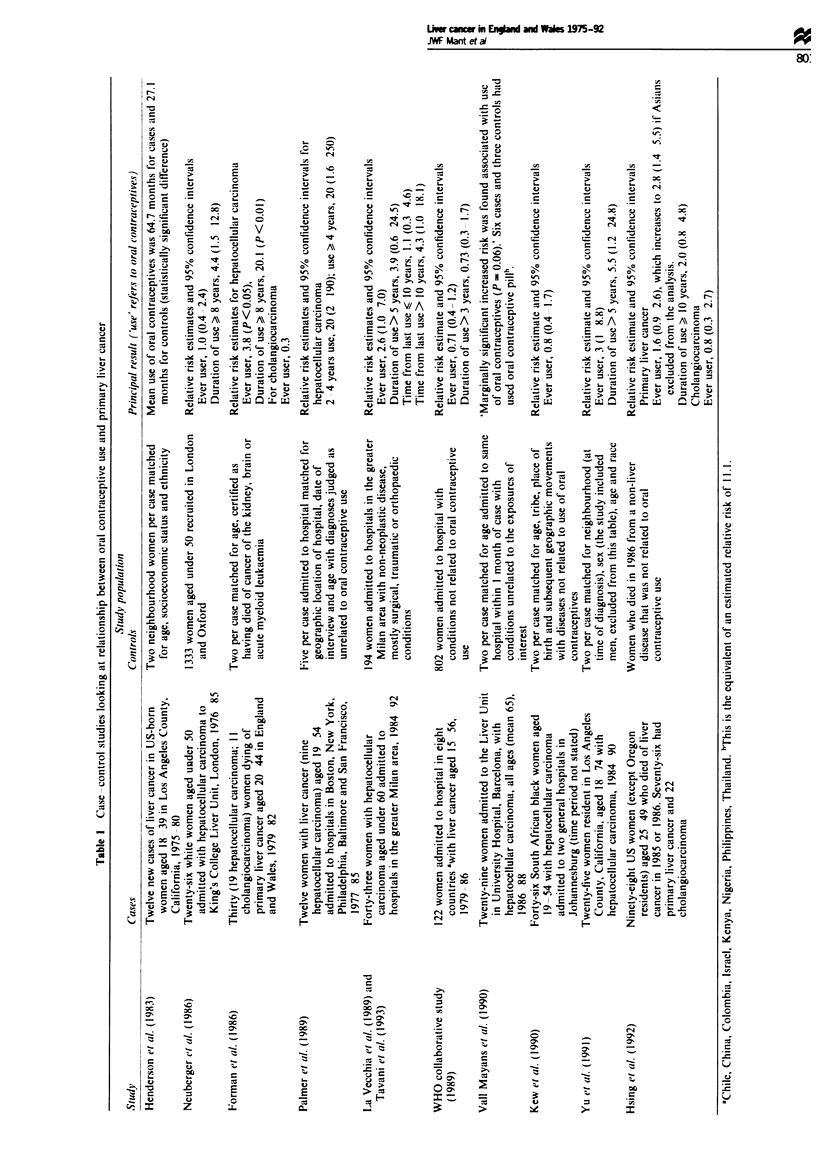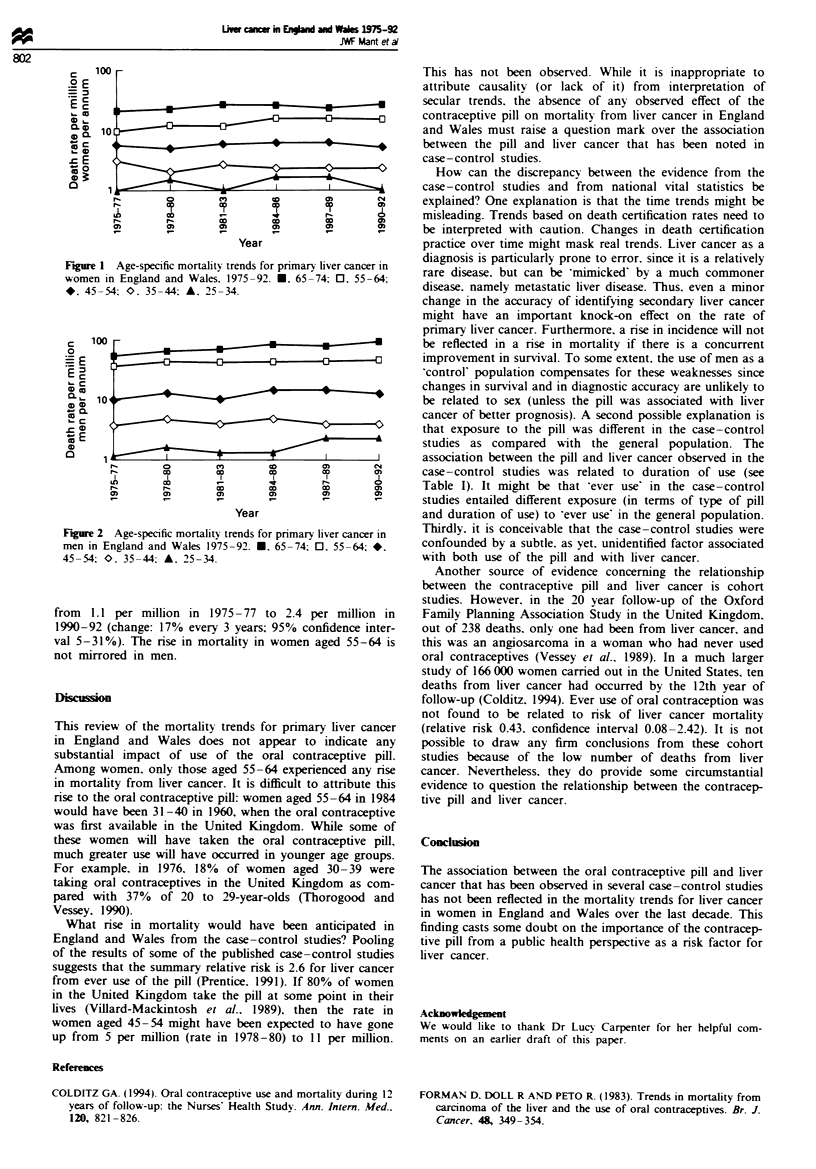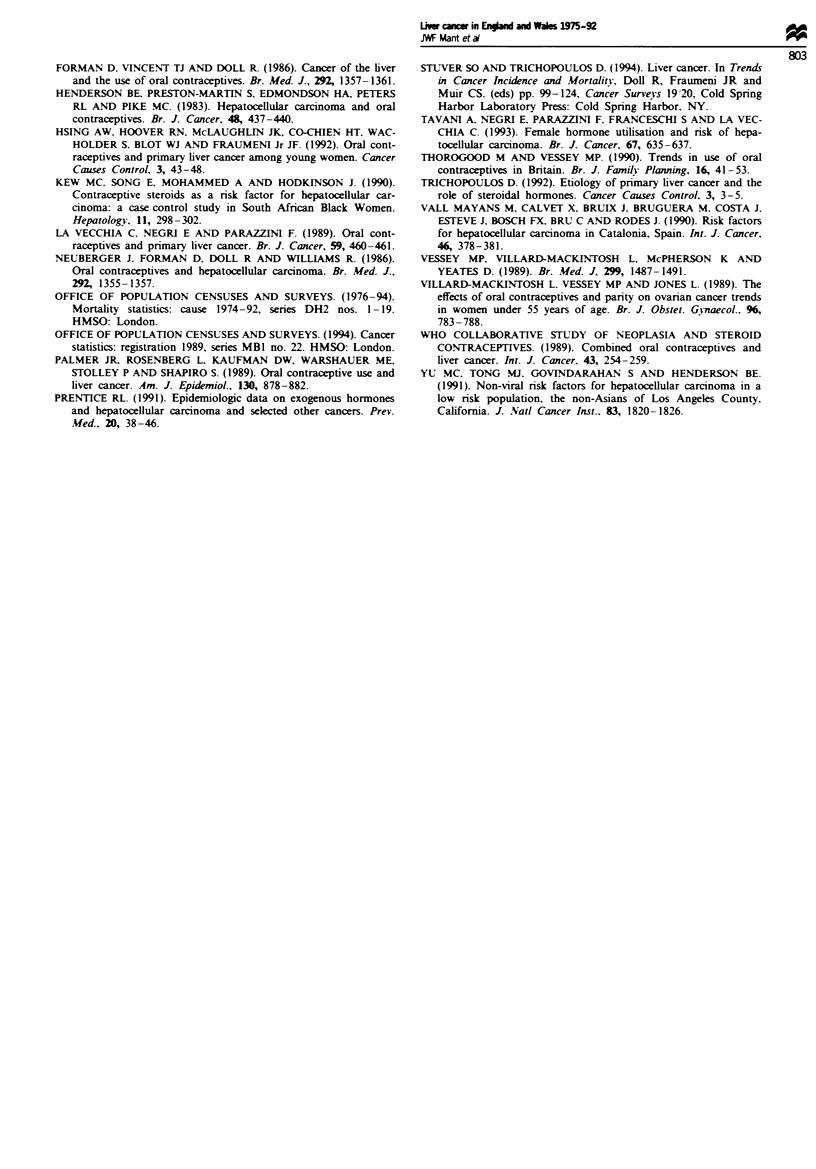# Trends in mortality from primary liver cancer in England and Wales 1975-92: influence of oral contraceptives.

**DOI:** 10.1038/bjc.1995.415

**Published:** 1995-09

**Authors:** J. W. Mant, M. P. Vessey

**Affiliations:** Department of Public Health and Primary Care, University of Oxford, Radcliffe Infirmary, UK.

## Abstract

Numerous case-control studies have suggested a link between the oral contraceptive pill and liver cancer. The secular trends in liver cancer mortality rates for England and Wales from 1975 to 1992 were examined to determine whether an effect of the pill was apparent. Liver cancer mortality has remained constant in women in the age groups that have had major exposure to the pill.


British Journal of Cancer (1995) 72, 800-803

© 1995 Stockton Press All rights reserved 0007-0920/95 $12.00

SHORT COMMUNICATION

### Trends in mortality from primary liver cancer in England and Wales 1975-92: influence of oral contraceptives

JWF Mant and MP Vessey

*Department of Public Health and Primary Care, University of Oxford, Gibson Building, Radcliffe Infirmary, Oxford OX2 6HE, UK.*

**Summary** Numerous case-control studies have suggested a link between the oral contraceptive pill and liver cancer. The secular trends in liver cancer mortality rates for England and Wales from 1975 to 1992 were examined to determine whether an effect of the pill was apparent. Liver cancer mortality has remained constant in women in the age groups that have had major exposure to the pill.

**Keywords:** mortality trend; hepatocellular carcinoma; oral contraceptive

Evidence is accumulating from case-control studies of an association between the use of oral contraceptives and hepatocellular carcinoma in countries where the incidence of hepatitis B is low. Table I summarises the ten such studies exploring this relationship that have been published over the last decade. With the exception of two studies (WHO, 1989; Kew *et al.*, 1990), the results point to a link between oral contraceptives and liver cancer, with risk increasing with duration of use (Forman *et al.*, 1986; Neuberger *et al.*, 1986; Yu *et al.*, 1991; Tavani *et al.*, 1993). There is also some evidence that the effect persists for over 10 years after cessation of use (Tavani *et al.*, 1993). In contrast to the other studies, the two which did not demonstrate any association with liver cancer were both in areas where hepatitis B is endemic. For example, in Kew *et al.*'s study of liver cancer in black South African women, 96% of patients were hepatitis B positive, compared with 62% of the controls (relative risk 13.5).

Internationally, variations in incidence of and mortality from primary liver cancer are difficult to interpret because of changes in classification and variations in diagnostic accuracy over time and in different parts of the world (Stuver and Trichopoulos, 1994). The most important aetiological factor for liver cancer world-wide may well be hepatitis B (Trichopoulos, 1992). However, in countries where the incidence of hepatitis B is low and liver cancer is correspondingly rare, it may be that use of the oral contraceptive pill is an important determinant of liver cancer mortality trends in women. Since the late 1960s, the contraceptive pill has been in widespread use in the United Kingdom, to the extent that over 80% of women born in the UK in the 1950s have had some experience of it (Villard-Mackintosh *et al.*, 1989; Thorogood and Vessey, 1990). The possible impact of the pill on mortality from liver cancer in England and Wales between 1958 and 1981 was explored by Forman *et al.* (1983) using national death certification rates. They found that there was indeed a statistically significant increase in the death rate of women aged 20-39 from 0.9 to 1.8 per million when comparing the death rate from 1970-75 with that from 1976-81. This increase was not found in men or older women. The aim of this paper is to review the mortality trends in England and Wales from primary liver cancer since Forman *et al.*'s analysis to observe whether this rise has been sustained.

Correspondence: J Mant
Received 8 December 1994; revised 21 March 1995; accepted 10 April 1995

### Materials and methods

National death certification rates for England and Wales for the period 1975 to 1992 were examined in 10 year age bands covering the age range 25-74 for both women and men (to give a 'control' population). Given the low incidence of the disease in England and Wales, the rates were aggregated over 3 year periods. The information was obtained from routinely published national statistics (Office of Population Censuses and Surveys, 1976-94). The numerator was restricted to deaths coded as 155.0 by the 8th (for 1975-78) and 9th (1979-92) revisions of the ICD codes - 'primary liver cancer'. Cholangiocarcinoma (155.1) was excluded since the case-control studies that had suggested a link with oral contraceptive use were largely concerned with hepatocellular carcinoma (see Table I). Those that did look for any association between the pill and cholangiocarcinoma have been inconclusive (Forman *et al.*, 1986; WHO, 1989; Hsing *et al.*, 1992). Cholangiocarcinoma and primary liver cancer are not separated out in the published cancer incidence data for England and Wales (OPCS, 1994), nor are they separated out in published mortality data before 1975. Therefore, the analysis is restricted to mortality data from 1975 to 1992.

Where a trend was observed, a point estimate for the trend was made and approximate 95% confidence intervals calculated using a poisson regression model on General Linear Interactive Modelling (GLIM) software.

### Results

Figure 1 shows the age-specific mortality trends for women from primary liver cancer over the period 1975-92. There have been no further rises in the death rate from liver cancer in young women since that observed by Forman *et al.* (1983). The rate in women aged 25-34 has stayed at around 1.4 per million over the study period. Similarly, there has been no discernible trend in women aged 35-44 or 45-54, with the annual death rates staying around 2.3 per million and 5.9 per million respectively. There was a rise in the death rate for women aged 55-64 from 10 per million in 1975-77 to 15 per million in 1990-92 (change: 10% rise every 3 years; 95% confidence interval 5-15%).

Figure 2 shows the trends for men over the same period. The death rates are much higher for each age group. There has been a change in the rate in 65 to 74-year-olds from 55 per million in 1975-77 to 96 per million in 1990-92 (change: 11% every 3 years; 95% confidence interval 8-13%). There has also been a rise in mortality in the 25 to 34-year-olds,

**Table 1** Case–control studies looking at relationship between oral contraceptive use and primary liver cancer

| Study | Study population: Cases | Study population: Controls | Principal result ('use' refers to oral contraceptives) |
|---|---|---|---|
| Henderson *et al.* (1983) | Twelve new cases of liver cancer in US-born women aged 18–39 in Los Angeles County, California, 1975–80 | Two neighbourhood women per case matched for age, socioeconomic status and ethnicity | Mean use of oral contraceptives was 64.7 months for cases and 27.1 months for controls (statistically significant difference) |
| Neuberger *et al.* (1986) | Twenty-six white women aged under 50 admitted with hepatocellular carcinoma to King's College Liver Unit, London, 1976–85 | 1333 women aged under 50 recruited in London and Oxford | Relative risk estimates and 95% confidence intervals<br>Ever user, 1.0 (0.4–2.4)<br>Duration of use $\geq$ 8 years, 4.4 (1.5–12.8) |
| Forman *et al.* (1986) | Thirty (19 hepatocellular carcinoma; 11 cholangiocarcinoma) women dying of primary liver cancer aged 20–44 in England and Wales, 1979–82 | Two per case matched for age, certified as having died of cancer of the kidney, brain or acute myeloid leukaemia | Relative risk estimates for hepatocellular carcinoma<br>Ever user, 3.8 ($P<0.05$),<br>Duration of use $\geq$ 8 years, 20.1 ($P<0.01$)<br>For cholangiocarcinoma<br>Ever user, 0.3 |
| Palmer *et al.* (1989) | Twelve women with liver cancer (nine hepatocellular carcinoma) aged 19–54 admitted to hospitals in Boston, New York, Philadelphia, Baltimore and San Francisco, 1977–85 | Five per case admitted to hospital matched for geographic location of hospital, date of interview and age with diagnoses judged as unrelated to oral contraceptive use | Relative risk estimates and 95% confidence intervals for hepatocellular carcinoma<br>2–4 years use, 20 (2–190); use $\geq$ 4 years, 20 (1.6–250) |
| La Vecchia *et al.* (1989) and Tavani *et al.* (1993) | Forty-three women with hepatocellular carcinoma aged under 60 admitted to hospitals in the greater Milan area, 1984–92 | 194 women admitted to hospitals in the greater Milan area with non-neoplastic disease, mostly surgical, traumatic or orthopaedic conditions | Relative risk estimates and 95% confidence intervals<br>Ever user, 2.6 (1.0–7.0)<br>Duration of use > 5 years, 3.9 (0.6–24.5)<br>Time from last use $\leq$ 10 years, 1.1 (0.3–4.6)<br>Time from last use > 10 years, 4.3 (1.0–18.1) |
| WHO collaborative study (1989) | 122 women admitted to hospital in eight countries [a]with liver cancer aged 15–56, 1979–86 | 802 women admitted to hospital with conditions not related to oral contraceptive use | Relative risk estimate and 95% confidence intervals<br>Ever user, 0.71 (0.4–1.2)<br>Duration of use > 3 years, 0.73 (0.3–1.7) |
| Vall Mayans *et al.* (1990) | Twenty-nine women admitted to the Liver Unit in University Hospital, Barcelona, with hepatocellular carcinoma, all ages (mean 65), 1986–88 | Two per case matched for age admitted to same hospital within 1 month of case with conditions unrelated to the exposures of interest | 'Marginally significant increased risk was found associated with use of oral contraceptives ($P = 0.06$).' Six cases and three controls had used oral contraceptive pill[b]. |
| Kew *et al.* (1990) | Forty-six South African black women aged 19–54 with hepatocellular carcinoma admitted to two general hospitals in Johannesburg (time period not stated) | Two per case matched for age, tribe, place of birth and subsequent geographic movements with diseases not related to use of oral contraceptives | Relative risk estimate and 95% confidence intervals<br>Ever user, 0.8 (0.4–1.7) |
| Yu *et al.* (1991) | Twenty-five women resident in Los Angeles County, California, aged 18–74 with hepatocellular carcinoma, 1984–90 | Two per case matched for neighbourhood (at time of diagnosis), sex (the study included men, excluded from this table), age and race | Relative risk estimate and 95% confidence intervals<br>Ever user, 3 (1–8.8)<br>Duration of use > 5 years, 5.5 (1.2–24.8) |
| Hsing *et al.* (1992) | Ninety-eight US women (except Oregon residents) aged 25–49 who died of liver cancer in 1985 or 1986. Seventy-six had primary liver cancer and 22 cholangiocarcinoma | Women who died in 1986 from a non-liver disease that was not related to oral contraceptive use | Relative risk estimate and 95% confidence intervals<br>Primary liver cancer<br>Ever user, 1.6 (0.9–2.6), which increases to 2.8 (1.4–5.5) if Asians excluded from the analysis.<br>Duration of use $\geq$ 10 years, 2.0 (0.8–4.8)<br>Cholangiocarcinoma<br>Ever user, 0.8 (0.3–2.7) |

[a]Chile, China, Colombia, Israel, Kenya, Nigeria, Philippines, Thailand. [b]This is the equivalent of an estimated relative risk of 11.1.

**Figure 1** Age-specific mortality trends for primary liver cancer in women in England and Wales, 1975-92. ■, 65-74; □, 55-64; ◆, 45-54; ◇, 35-44; ▲, 25-34.

**Figure 2** Age-specific mortality trends for primary liver cancer in men in England and Wales 1975-92. ■, 65-74; □, 55-64; ◆, 45-54; ◇, 35-44; ▲, 25-34.

from 1.1 per million in 1975-77 to 2.4 per million in 1990-92 (change: 17% every 3 years; 95% confidence interval 5-31%). The rise in mortality in women aged 55-64 is not mirrored in men.

### Discussion

This review of the mortality trends for primary liver cancer in England and Wales does not appear to indicate any substantial impact of use of the oral contraceptive pill. Among women, only those aged 55-64 experienced any rise in mortality from liver cancer. It is difficult to attribute this rise to the oral contraceptive pill: women aged 55-64 in 1984 would have been 31-40 in 1960, when the oral contraceptive was first available in the United Kingdom. While some of these women will have taken the oral contraceptive pill, much greater use will have occurred in younger age groups. For example, in 1976, 18% of women aged 30-39 were taking oral contraceptives in the United Kingdom as compared with 37% of 20 to 29-year-olds (Thorogood and Vessey, 1990).

What rise in mortality would have been anticipated in England and Wales from the case-control studies? Pooling of the results of some of the published case-control studies suggests that the summary relative risk is 2.6 for liver cancer from ever use of the pill (Prentice, 1991). If 80% of women in the United Kingdom take the pill at some point in their lives (Villard-Mackintosh *et al.*, 1989), then the rate in women aged 45-54 might have been expected to have gone up from 5 per million (rate in 1978-80) to 11 per million. This has not been observed. While it is inappropriate to attribute causality (or lack of it) from interpretation of secular trends, the absence of any observed effect of the contraceptive pill on mortality from liver cancer in England and Wales must raise a question mark over the association between the pill and liver cancer that has been noted in case-control studies.

How can the discrepancy between the evidence from the case-control studies and from national vital statistics be explained? One explanation is that the time trends might be misleading. Trends based on death certification rates need to be interpreted with caution. Changes in death certification practice over time might mask real trends. Liver cancer as a diagnosis is particularly prone to error, since it is a relatively rare disease, but can be 'mimicked' by a much commoner disease, namely metastatic liver disease. Thus, even a minor change in the accuracy of identifying secondary liver cancer might have an important knock-on effect on the rate of primary liver cancer. Furthermore, a rise in incidence will not be reflected in a rise in mortality if there is a concurrent improvement in survival. To some extent, the use of men as a 'control' population compensates for these weaknesses since changes in survival and in diagnostic accuracy are unlikely to be related to sex (unless the pill was associated with liver cancer of better prognosis). A second possible explanation is that exposure to the pill was different in the case-control studies as compared with the general population. The association between the pill and liver cancer observed in the case-control studies was related to duration of use (see Table I). It might be that 'ever use' in the case-control studies entailed different exposure (in terms of type of pill and duration of use) to 'ever use' in the general population. Thirdly, it is conceivable that the case-control studies were confounded by a subtle, as yet, unidentified factor associated with both use of the pill and with liver cancer.

Another source of evidence concerning the relationship between the contraceptive pill and liver cancer is cohort studies. However, in the 20 year follow-up of the Oxford Family Planning Association Study in the United Kingdom, out of 238 deaths, only one had been from liver cancer, and this was an angiosarcoma in a woman who had never used oral contraceptives (Vessey *et al.*, 1989). In a much larger study of 166 000 women carried out in the United States, ten deaths from liver cancer had occurred by the 12th year of follow-up (Colditz, 1994). Ever use of oral contraception was not found to be related to risk of liver cancer mortality (relative risk 0.43, confidence interval 0.08-2.42). It is not possible to draw any firm conclusions from these cohort studies because of the low number of deaths from liver cancer. Nevertheless, they do provide some circumstantial evidence to question the relationship between the contraceptive pill and liver cancer.

### Conclusion

The association between the oral contraceptive pill and liver cancer that has been observed in several case-control studies has not been reflected in the mortality trends for liver cancer in women in England and Wales over the last decade. This finding casts some doubt on the importance of the contraceptive pill from a public health perspective as a risk factor for liver cancer.

**Acknowledgement**
We would like to thank Dr Lucy Carpenter for her helpful comments on an earlier draft of this paper.

### References

COLDITZ GA. (1994). Oral contraceptive use and mortality during 12 years of follow-up: the Nurses' Health Study. *Ann. Intern. Med.*, **120**, 821-826.

FORMAN D, DOLL R AND PETO R. (1983). Trends in mortality from carcinoma of the liver and the use of oral contraceptives. *Br. J. Cancer*, **48**, 349-354.

FORMAN D, VINCENT TJ AND DOLL R. (1986). Cancer of the liver and the use of oral contraceptives. *Br. Med. J.*, **292**, 1357–1361.

HENDERSON BE, PRESTON-MARTIN S, EDMONDSON HA, PETERS RL AND PIKE MC. (1983). Hepatocellular carcinoma and oral contraceptives. *Br. J. Cancer*, **48**, 437–440.

HSING AW, HOOVER RN, McLAUGHLIN JK, CO-CHIEN HT, WACHOLDER S, BLOT WJ AND FRAUMENI Jr JF. (1992). Oral contraceptives and primary liver cancer among young women. *Cancer Causes Control*, **3**, 43–48.

KEW MC, SONG E, MOHAMMED A AND HODKINSON J. (1990). Contraceptive steroids as a risk factor for hepatocellular carcinoma: a case/control study in South African Black Women. *Hepatology*, **11**, 298–302.

LA VECCHIA C, NEGRI E AND PARAZZINI F. (1989). Oral contraceptives and primary liver cancer. *Br. J. Cancer*, **59**, 460–461.

NEUBERGER J, FORMAN D, DOLL R AND WILLIAMS R. (1986). Oral contraceptives and hepatocellular carcinoma. *Br. Med. J.*, **292**, 1355–1357.

OFFICE OF POPULATION CENSUSES AND SURVEYS. (1976–94). Mortality statistics: cause 1974–92, series DH2 nos. 1–19. HMSO: London.

OFFICE OF POPULATION CENSUSES AND SURVEYS. (1994). Cancer statistics: registration 1989, series MB1 no. 22. HMSO: London.

PALMER JR, ROSENBERG L, KAUFMAN DW, WARSHAUER ME, STOLLEY P AND SHAPIRO S. (1989). Oral contraceptive use and liver cancer. *Am. J. Epidemiol.*, **130**, 878–882.

PRENTICE RL. (1991). Epidemiologic data on exogenous hormones and hepatocellular carcinoma and selected other cancers. *Prev. Med.*, **20**, 38–46.

STUVER SO AND TRICHOPOULOS D. (1994). Liver cancer. In *Trends in Cancer Incidence and Mortality*, Doll R, Fraumeni JR and Muir CS. (eds) pp. 99–124, *Cancer Surveys* 19/20, Cold Spring Harbor Laboratory Press: Cold Spring Harbor, NY.

TAVANI A, NEGRI E, PARAZZINI F, FRANCESCHI S AND LA VECCHIA C. (1993). Female hormone utilisation and risk of hepatocellular carcinoma. *Br. J. Cancer*, **67**, 635–637.

THOROGOOD M AND VESSEY MP. (1990). Trends in use of oral contraceptives in Britain. *Br. J. Family Planning*, **16**, 41–53.

TRICHOPOULOS D. (1992). Etiology of primary liver cancer and the role of steroidal hormones. *Cancer Causes Control*, **3**, 3–5.

VALL MAYANS M, CALVET X, BRUIX J, BRUGUERA M, COSTA J, ESTEVE J, BOSCH FX, BRU C AND RODES J. (1990). Risk factors for hepatocellular carcinoma in Catalonia, Spain. *Int. J. Cancer*, **46**, 378–381.

VESSEY MP, VILLARD-MACKINTOSH L, McPHERSON K AND YEATES D. (1989). *Br. Med. J*, **299**, 1487–1491.

VILLARD-MACKINTOSH L, VESSEY MP AND JONES L. (1989). The effects of oral contraceptives and parity on ovarian cancer trends in women under 55 years of age. *Br. J. Obstet. Gynaecol.*, **96**, 783–788.

WHO COLLABORATIVE STUDY OF NEOPLASIA AND STEROID CONTRACEPTIVES. (1989). Combined oral contraceptives and liver cancer. *Int. J. Cancer*, **43**, 254–259.

YU MC, TONG MJ, GOVINDARAHAN S AND HENDERSON BE. (1991). Non-viral risk factors for hepatocellular carcinoma in a low risk population, the non-Asians of Los Angeles County, California. *J. Natl Cancer Inst.*, **83**, 1820–1826.